# Effects of Doxycycline-Loaded NO-Releasing Nanomatrix Gel on Delayed Replanted of Rat Molar

**DOI:** 10.3390/gels10040213

**Published:** 2024-03-22

**Authors:** Mi Ja Ko, Mi Sun Kim, Hyo-Seol Lee, Ok Hyung Nam, Yong Kwon Chae, Sung Chul Choi

**Affiliations:** 1 Children Loving Dental Clinic, Seosan 31978, Republic of Korea; angelko2@naver.com; 2 Department of Pediatric Dentistry, College of Dentistry, Kyung Hee University, Kyung Hee University Hospital at Gangdong, Seoul 05278, Republic of Korea; pedokms@khu.ac.kr; 3 Department of Pediatric Dentistry, College of Dentistry, Kyung Hee University, Kyung Hee University Medical Center, Seoul 02447, Republic of Korea; stberryfield@gmail.com (H.-S.L.);; 4 Department of Pediatric Dentistry, Kyung Hee University Medical Center, Seoul 02447, Republic of Korea; pedochae@gmail.com

**Keywords:** avulsion, delayed replantation, nitric oxide, nanomatrix gel, doxycycline, NaF, tooth replantation

## Abstract

Background/Aim: Tooth avulsion and delayed replantation may cause inflammatory responses and root resorption of the tooth. The aim of this study is to investigate the effect of a doxycycline-loaded nitric oxide-releasing nanomatrix (DN) gel on the delayed replantation of avulsed rat teeth, with a focus on assessing the gel’s potential to promote regeneration and inhibit complications associated with the replantation process. Materials and Methods: Twenty-four right maxillary first molars from male Sprague-Dawley rats were atraumatically extracted using sterile extraction forceps. The molars were dried for 1 h at room temperature (approximately 23 °C) and divided into four groups according to the root conditioning methods after extra-alveolar 60-min drying: Group 1, no root conditioning treatment prior to replantation; Group 2, soaking in 2% NaF solution for 5 min before replantation; Group 3, 5-min soaking in NO gel and injection of the gel into the alveolar socket; Group 4, 5-min soaking in DN gel and injection of the gel into the alveolar socket before replantation. The animals were euthanized four weeks after the operation and the specimens were evaluated histologically. Results: The use of NO gel alone showed better anti-inflammatory and periodontal effects than the control group, but it did not show a significant effect compared to the group using NaF. When using NO gel loaded with doxycycline, it showed a significant anti-inflammatory effect compared to the control group and showed a similar inhibitory effect to the group using NaF. Conclusions: Within the limits of this study, in delayed replantation situations, the control of inflammatory resorption and replacement resorption is an important factor for achieving a better prognosis of replanted teeth. Root surface treatment with DN gel decreased root resorption after delayed replantation.

## 1. Introduction

Tooth avulsion is a complex and serious form of injury that occurs when a tooth is completely knocked out of its socket [1]. An avulsed tooth usually does not recover its function due to severe damage to the tooth and surrounding tissues [2,3]. The best prognosis for tooth replantation can be achieved when the extra-alveolar time does not exceed 5 min [4]. However, immediate replantation can be challenging due to the distressing situation for both patients and guardians, as well as a lack of knowledge about different trauma conditions and replantation techniques [5]. 

Research on the delayed replantation of avulsed teeth has primarily focused on preventing inflammatory root resorption and reducing inflammation in order to promote the regeneration and reattachment of periodontal ligament (PDL) cells [5,6,7,8]. This is because tooth replantation can lead to complications such as infection, pulp necrosis, apical periodontitis, and inflammatory or replacement root resorption, which may ultimately result in tooth loss [6]. For tooth replantation that has been delayed for more than 60 min, the application of a 2% sodium fluoride (NaF) solution is recommended to harden the root surface and prevent osteoclastic root resorption [6,9,10]. Another treatment method for delayed replantation is the application of bisphosphonate on the root surface [5,11,12]. The main mechanism of action of these drugs has been attributed to the inhibition of osteoclasts by decreasing osteoclast activity and the differentiation of monocytes and macrophages into osteoclasts [13].

Recent advances in regenerative tissue engineering have enabled the recovery of functional components of the original tissue and the structural replacement of defects in oral and craniofacial tissue by controlling the extra-cellular microenvironment [14,15,16]. Regenerative tissue engineering approaches are based on the tissue engineering triad: cells, their extra-cellular matrix (ECM), and signals, which can be used individually or in combination to optimize the regeneration and engineering of functional tissues [15].

Nitric oxide (NO) is a vital signaling molecule that plays a crucial role in controlling various physiological responses in the human body. During acute inflammation, NO helps regulate the progression of the inflammatory process, while in chronic inflammation, it promotes angiogenesis. Additionally, NO possesses potent antibacterial and antiviral properties that make it a promising therapeutic agent [17,18,19,20,21]. However, the clinical use of NO is challenging due to its short half-life, which necessitates the development of suitable delivery systems to facilitate its application [20,21].

A biomimetic nanomatrix gel is formed by self-assembled peptide amphiphiles (PAs), which consist of a hydrophilic functional peptide sequence attached to a hydrophobic alkyl tail. Numerous applications have been proposed for PAs, including heterogeneous catalysis, nanoelectronics, drug delivery, and tissue engineering [22]. In addition, they can form viscoelastic gels by themselves under certain circumstances. This gel can be used to trap and/or isolate cells, support cell growth, and provide a controlled environment that mimics the natural ECM [22,23]. Several studies have reported the clinical potential of PAs as drug delivery systems by both preventing inflammatory responses and promoting vasodilation [24,25,26]. 

Although several studies on the treatment of delayed avulsed teeth have been published in the last few decades, none have mentioned drug delivery systems. They have only focused on the application of specific drug (such as NaF, antibiotics, dexamethasone) in the root surface of avulsed tooth. On the basis of previous findings, we developed a doxycycline-loaded NO-releasing nanomatrix (DN) gel to inhibit complications and promote the regeneration of a replanted avulsed tooth [27]. DN gel exerts its regenerative effects via a carrier for the delivery of antibiotics and has potential in stem/precursor cell recruitment. The aim of this study was to investigate the effect of a doxycycline-loaded nitric oxide-releasing nanomatrix (DN) gel on the delayed replantation of avulsed rat teeth, with a focus on assessing the gel’s potential to promote regeneration and inhibit complications associated with the replantation process.

## 2. Results and Discussions

### 2.1. Histomorphometry Analysis of the Experimental Group

All specimens exhibited various types of periodontal responses. A histomorphometric analysis was performed using a light microscope. Descriptive microscopic analysis of the sections was performed as follows.

(1) Group 1 (no treatment)

**Figure 1:**
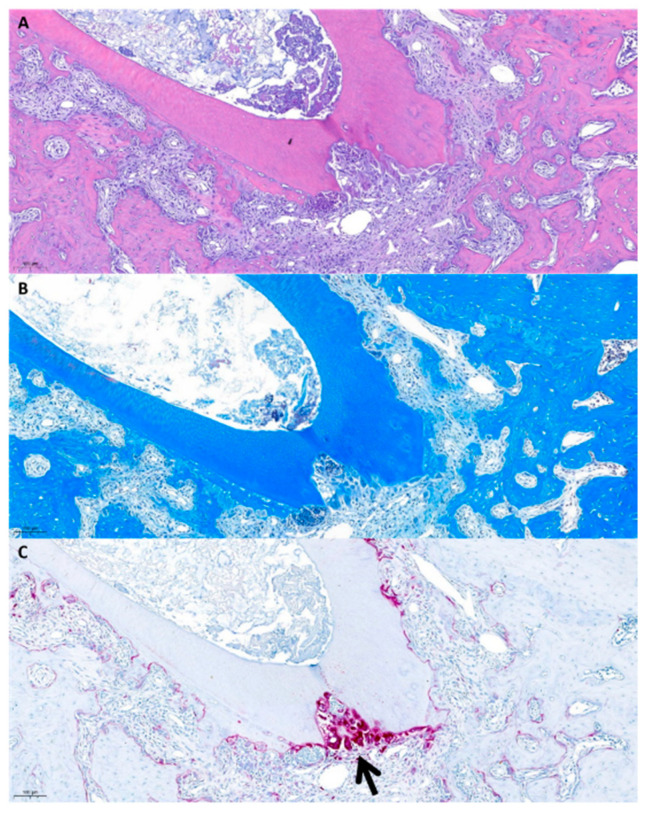
Histological photomicrographs of Group 1. (**A**) H&E stains, (**B**) MT stains, and (**C**) TRAP stains. The arrow shows intensive osteoclasts in the resorption area of the root.

The control group (without root surface treatment) showed inflammatory resorption in the replanted teeth, and replacement resorption appeared to be less frequent (Figure 1). In the H&E staining photomicrograph, the infiltration of inflammatory cells was observed in the apical resorbed area (Figure 1A), and direct tooth-to-bone contact was observed in the H&E and MT staining photomicrographs (Figure 1A,B). In TRAP staining photomicrographs, osteoclasts appeared intensively in the resorbing area of the root surface (Figure 1C).

(2) Group 2 (2% NaF)

**Figure 2:**
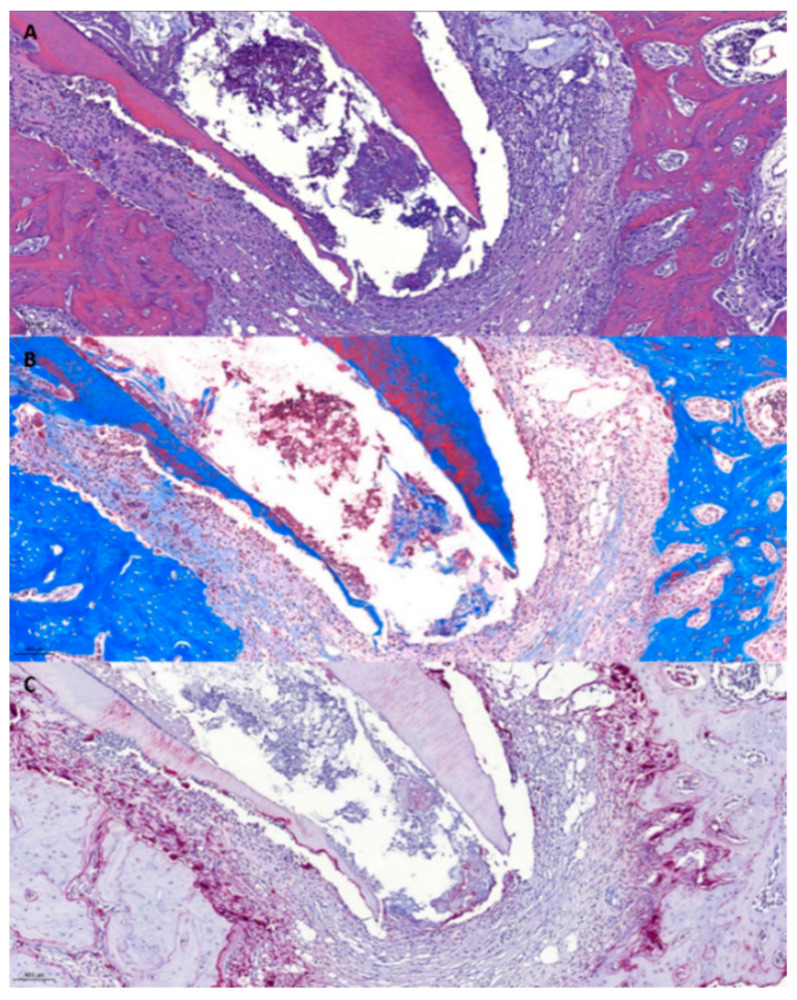
Histological photomicrographs of Group 2. (**A**) H&E stains. Inflammatory cells are observed. (**B**) MT stains. (**C**) TRAP stains.

In the H&E staining photomicrograph, the infiltration of inflammatory cells around the root was observed (Figure 2A). An inflammatory response was also shown in the MT staining photomicrograph (Figure 2B). In the TRAP staining photomicrograph, alveolar bone resorption was different from that observed in periodontitis (Figure 2C).

(3) Group 3 (NO gel)

**Figure 3:**
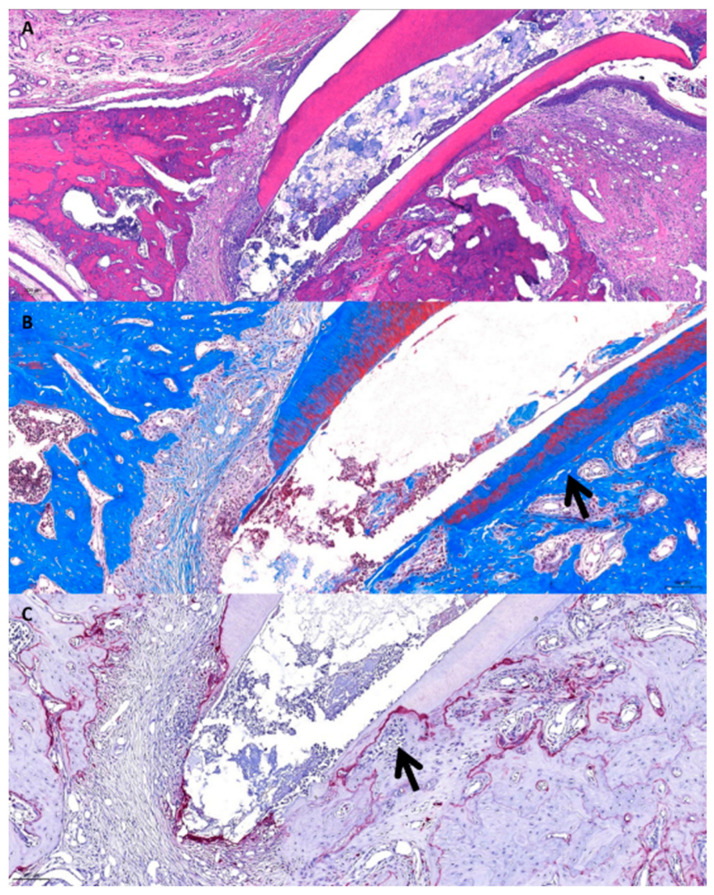
Histological photomicrographs of Group 3. (**A**) H&E stains. (**B**) MT stains. The arrow shows the initial stage of the displacement resorption. (**C**) TRAP stains. The arrow shows concentrated osteoclasts in the displacement resorption area.

Resorption at the apical quarter and replacement resorption were observed together (Figure 3A). MT staining photomicrographs showed the initial stage of displacement resorption. The blue-stained area showed that the roots and bones were directly connected (Figure 3B). The TRAP staining photomicrograph showed concentrated active odontoclasts in the displacement resorption area (Figure 3C).

(4) Group 4 (DN gel)

**Figure 4:**
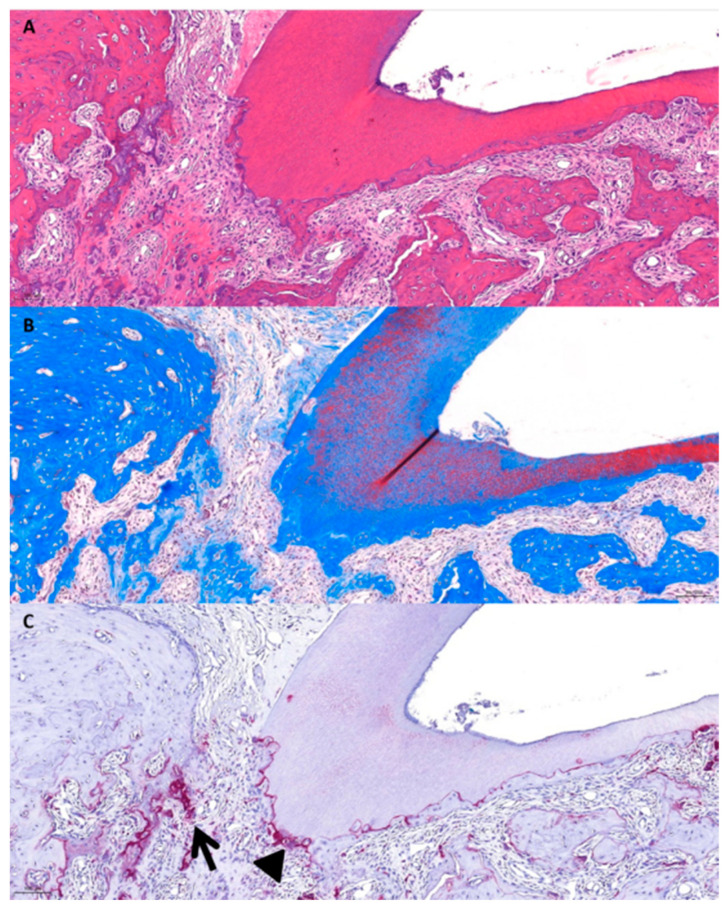
Histological photomicrographs of Group 4. (**A**) H&E stains. (**B**) MT stains. (**C**) TRAP stains. The arrow shows slight osteoclasts in the resorption area of the root and the triangle indicates activated osteoclasts.

Localized root resorption was observed from the apical region to the dentin region, beyond the cementum region (Figure 4). Inflammatory responses occurred superficially, and periodontal fibers were observed to be normally arranged (Figure 4A,B). In addition, the TRAP staining photomicrograph showed that osteoclasts were slightly present on the root surface and could be seen in areas of bone remodeling (Figure 4C).

### 2.2. Effects on Periodontal Healing

**Figure 5:**
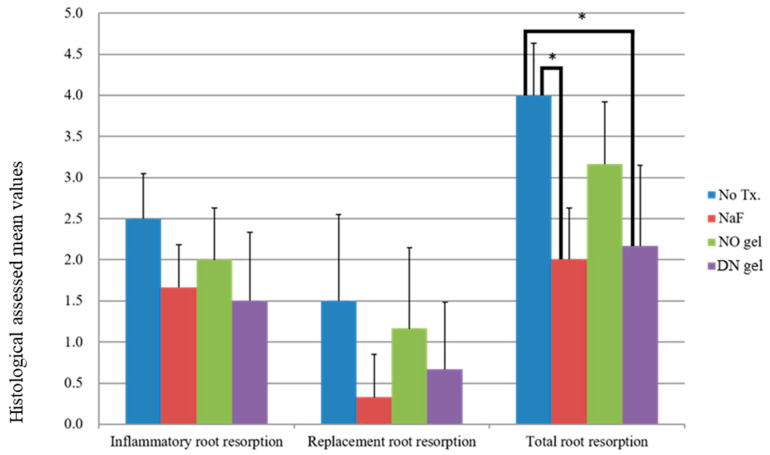
Results of the histological analyses of the periodontal healing process according to groups (* *p* < 0.05).

In periodontal analysis, all groups with root conditioning showed favorable PDL healing compared with the no-treatment group; however, there was no statistically significant difference in the results of inflammation and replacement root resorptions between the experimental groups. In total root resorption, Group 2 (NaF) and Group 4 (DN gel) showed a significantly lower grade than the control group (Table 1). Figure 5 demonstrates histological results.

### 2.3. Discussions

Tooth avulsion is the most serious type of dental injury. Studies have shown that the clinical success rate of avulsion is poor (ranging from 4% to 50%) [28]. Predicting the prognosis of replanted teeth is very complex and depends on the state of the avulsed teeth, PDL tissues, and supported alveolar bone. If the replantation of an avulsed tooth is delayed, unfavorable outcomes are highly likely to occur. Therefore, the treatment focus for avulsed teeth is to prevent inflammatory root resorption or reduce inflammatory responses to ensure periodontal regeneration [4,28,29].

For successful healing after replanting avulsed teeth, PDL cells must be preserved, and the state of PDL cells differs depending on the extra-oral time, storage medium, and contamination [30,31]. Many researchers and clinicians are conducting studies and developing treatment methods to save avulsed teeth; however, the most important thing is to keep the dislocated tooth in an anti-inflammatory condition. A good storage medium for avulsed teeth should be similar to the oral environment, i.e., an environment that has antimicrobial characteristics, can preserve the viability of cellular PDL, and is easy to use and access [32,33,34].

The viability of the PDL of the replanted tooth is the most important factor in determining the prognosis. When the extra-alveolar time of the avulsed tooth is >20 min, the PDL begins to necrose, and the risk of inflammation and root resorption increases in proportion to the extra-oral dry time when the tooth is replanted. Given that the extra-oral time is over 1 h, resorption along the whole root surface is expected [35,36].

In particular, when the extra-alveolar time is over 1 h, the presence of contaminated and necrotized remnants around the root surface becomes responsible for the increased occurrence of root resorption. Therefore, the treatment of avulsed teeth that have been extra-oral for more than 1 h has focused on rendering the root more resistant to resorption [37,38].

Avulsion causes the rupture of the neurovascular bundles, thus leading to pulp necrosis and bacterial contamination. Therefore, root canal treatment is recommended for avulsed teeth [39,40]. An association between bacteria embedded in the root canal system or dentinal tubules and cementum/PDL injury triggers inflammatory root resorption [41,42]. 

PDL necrosis results in the loss of important structures, such as the epithelial rests of Malassez, precementum, and cementoblasts, which appear to play an important role in the preservation of the PDL space [43]. Therefore, necrotic PDL remnants attached to the roots are mechanically removed as a part of the root surface treatment to preserve the cementum layer [44]. 

Another route of inflammation other than pulp necrosis is root surface exposure to the environment during the extra-alveolar period. In this case, the infection can be controlled using two methods: systemic antibiotic therapy and root surface treatment with various substances [42]. All animals in this study received intramuscular injections of penicillin G antibiotic. 

To date, various substances such as Emdogain^®^, enamel matrix derivatives, dexamethasone, bisphosphonate, tetracycline, and vitamin C have been used for root surface treatment [6,45,46,47,48]. In this study, we evaluated whether DN gel could prevent inflammatory root resorption in patients with delayed replantation. In addition, we investigated the therapeutic effect of NO gel alone and examined the differences with or without the addition of antibiotics. NO gel has been reported to inhibit the apoptosis of vascular endothelial cells and has anti-inflammatory effects [49]; therefore, it was expected to play an important role in the healing process of replanted teeth. In our previous studies, NO gel was found to be helpful in maintaining root growth and regeneration [27,28].

The topical administration of antibiotics at the time of replantation has been reported to reduce the frequency of inflammatory changes [50]. Topical treatment with doxycycline reduces microorganisms not only in the pulpal lumen but also on the root surface; therefore, this type of treatment could decrease the frequency of ankylosis [51]. Additionally, doxycycline inhibits the activity of matrix metalloproteinases (MMPs), which are the key enzymes involved in periodontal tissue destruction [52,53]. A previous study demonstrated that the expression of MMP-1 and MMP-8 after replantation increased with extra-oral time in avulsed rat teeth [54]. In addition, doxycycline exerts anti-inflammatory effects by inhibiting RANKL-induced osteoclastogenesis [55]. We believe that previous studies support our results regarding the inflammatory inhibitory responses shown in our experimental groups.

A 2% NaF solution has been widely used for root surface treatment and has shown good results for the delayed implantation of teeth [46,56]. Fluoride acts on cementum and dentin to convert hydroxyapatite to fluorapatite to make them more resistant to resorption and inhibit clastic cell formation [56]. In the current study, the drug was soaked for the same time to avoid any differences between groups, and we believe that additional experiments with various soaking times will be needed in the future.

NO inhibits the apoptosis of vascular endothelial cells induced by proinflammatory cytokines and proatherosclerotic factors, including angiotensin II. Endothelial cell apoptosis may contribute to the development of inflammation. Thus, NO can inhibit the inflammatory processes [49]. In our results, Group 3 (NO gel) showed slightly decreased inflammatory root resorption, replacement root resorption, and total root resorption compared with those in the control group.

DN gel decreased inflammatory root resorption, replacement root resorption, and total root resorption compared with the control group. The results of this study indicate that root canal treatment and systemic antibiotic treatment were similar in all groups, and inflammatory resorption was more frequent in Group 1; therefore, the root surface treatment of extracted teeth made a difference in the results.

In a previous study, an antibiotic-releasing nanomatrix gel demonstrated synergistic antibacterial effects [57]. Their results showed that NO gel had a greater synergistic antibacterial effect than antibiotics alone. The exact reason for the synergistic effect was not elucidated, but one of their hypotheses might be that the PA portion of the NO gel encapsulated the antibiotic, thus promoting the sustained local release of the antibiotic and resulting in better results. The current study also showed similar results to this previous study. The exact drug release kinetic test was not conducted in this experiment, but it has been known that many kinds of scaffolds have been used for the sustained and controlled release of drugs [58]. 

While this study had some limitations, including the use of only H&E and MT staining for quantitative analysis and the inability to perform quantitative analysis with TRAP staining due to variations in inflammation and time, new analyzing methods such as digital image analysis could be employed in future studies. Additionally, research is ongoing to explore novel treatment methods for delayed replantation, such as the use of regenerative therapies involving stem cells, growth factors, and bioactive materials to enhance periodontal regeneration and promote root surface healing.

## 3. Conclusions

Within the limit of this study, in delayed replantation situations, the control of inflammatory resorption and replacement resorption is an important factor for achieving a better prognosis of replanted teeth. Root surface treatment with DN gel decreased root resorption after delayed replantation. Further studies with larger sample sizes are warranted.

## 4. Materials and Methods

The experimental protocol was reviewed and approved by the Institutional Animal Care and Use Committee of Kyung Hee Medical Center, Kyung Hee University, Seoul, Republic of Korea (KHMC-IACUC-18-025).

### 4.1. Preparation of the DN Gel

**Figure 6:**
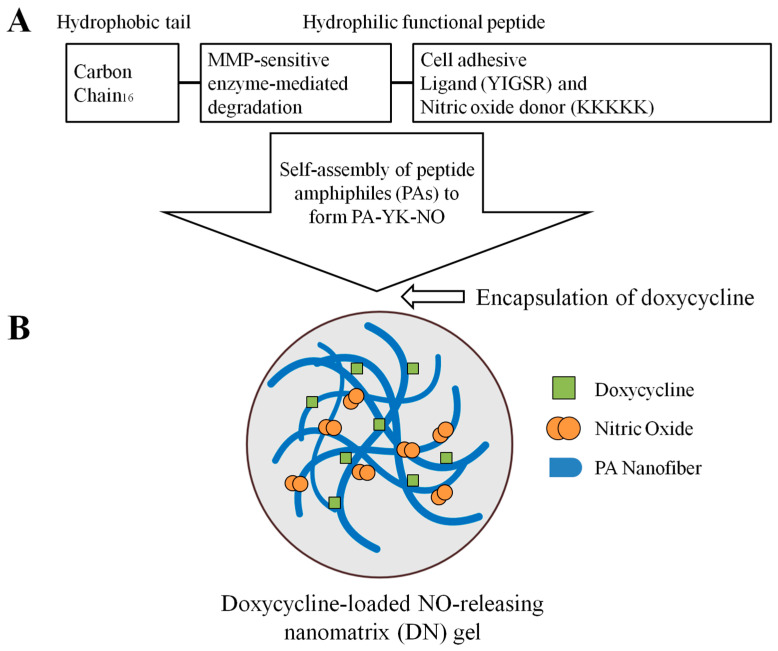
Schematic of doxycycline-loaded NO-releasing nanomatrix (DN) gel. (**A**) Self-assembly of peptide amphiphiles (PAs) to form PA-YK-NO. (**B**) Encapsulation of doxycycline.

The experiment was performed using a PA-YK (Tyrosine-Lysine)-NO gel, which was previously used in the experiment [23,32]. An endothelial cell adhesive ligand (YIGSR, tyrosine-isoleucine-glycine-serine-arginine) coupled with a matrix metalloprotease-2 (MMP-2) degradable sequence (GTAGLIGQ) formed PA-YIGSR. NO donor KKKKK (Poly lysine) linked to the MMP-2 degradable sequence formed PA-KKKKK. A mixture of PA-YIGSR and PA-KKKKK at a 9:1 molar ratio was reacted with NO gas to generate PA-YK-NO. Thereafter, gelation was achieved by mixing 50 μL of a 2 wt% PA-YK-NO solution with 15 μL 1 M calcium chloride and 25 μL phosphate-buffered saline (1X PBS, 137 mM NaCl, 10 mM phosphate and 2.7 mM KCl with pH 7.4), followed by incubation at 37 °C for 30 min. In a previous study [32], doxycycline was successfully encapsulated in a biomimetic nanomatrix gel. To adjust the viscosity, 10 mg antibiotic powder of doxycycline was dissolved in 1 mL propylene glycol. Figure 6 illustrates a schematic of the DN gel.

### 4.2. Animal Preparation and Replantation Procedures

#### 4.2.1. Preparing Animals and Defining Experimental Groups

Twenty-four male Sprague-Dawley rats (Samtako, Gyeonggi-Do, Republic of Korea) aged six to eight weeks and approximately 200–300 g in weight were used in this study. During the experimental period, the animals were fed a soft diet and provided an ample supply of water. They were divided into four groups according to the root conditioning methods after extra-alveolar 60-min drying: Group 1, no root conditioning treatment prior to replantation; Group 2, soaking in 2% NaF solution for 5 min before replantation; Group 3, 5-min soaking in NO gel and injection of the gel into the alveolar socket; Group 4, 5-min soaking in DN gel and injection of the gel into the alveolar socket before replantation. The experimental groups are presented in Table 2.

#### 4.2.2. Surgical Procedures and Root Conditioning

For all procedures, the animals were given a five-day supply of 0.4% β-aminopropionitrile (Sigma-Aldrich, St Louis, MO, USA) to avoid traumatic extraction. They were anesthetized with an intraperitoneal injection of Zoletil 50 (100–150 mg/kg; Virbac Lab, Carros, France). After cleaning the oral cavity with 2% chlorhexidine solution, 24 right maxillary first molars from 24 rats were atraumatically extracted using sterile extraction forceps. Any fractured root during the extraction procedure was excluded, and experimental animals were recruited for substitutions. Root canal treatment was performed outside the oral cavity to reduce the risk of inflammation in the rat molars. To minimize pulpal infection as a stimulus for external root resorption, all teeth were accessed, and the mesiobuccal canal was instrumented with K-type files to the apical stop and irrigated with sterile saline. The canal was dried using paper points and obturated using Vitapex (Neo Dental Chemical Products Co., Ltd., Tokyo, Japan). Occlusal accesses were filled with Caviton (GC Co., Tokyo, Japan). Thereafter, the molars were placed in a cell-culturing 24-well plate and then dried for 1 h at room temperature (approximately 23 °C).

**Figure 7:**
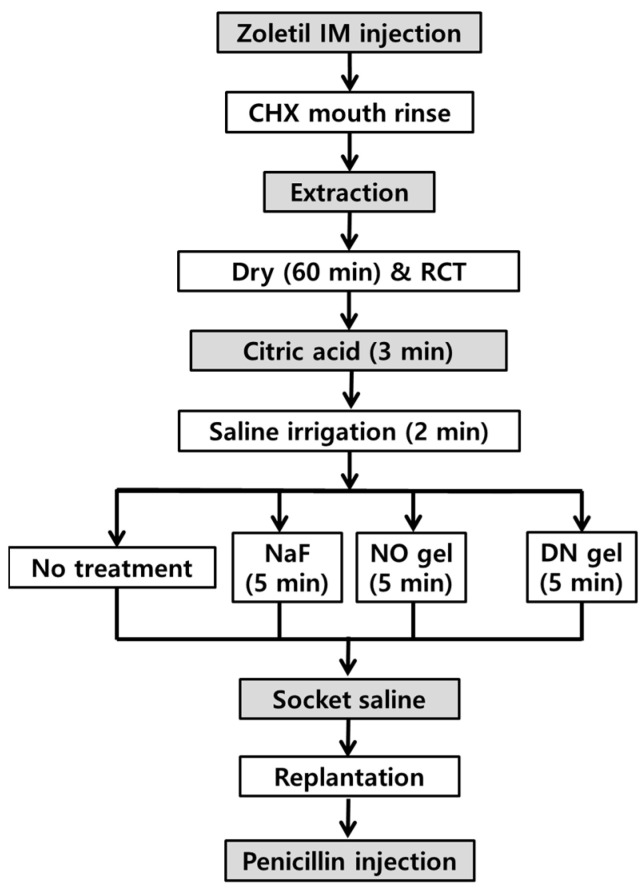
Flowchart of the experimental groups and procedures.

The tooth was dried at room temperature, followed by placement in citric acid for 3 min, curettage, and cleaning with saline for 2 min to remove impaired and dead periodontal tissues. A root conditioning agent was then applied to the molars for each group and gently replanted. After replantation, all animals received a single intramuscular injection of 20,000 IU of penicillin G (Alvogen potassium penicillin G, Alvogen, Seoul, Republic of Korea), and were fed a soft diet for seven days. The flowchart of the experimental procedure is presented in Figure 7.

### 4.3. Histological Evaluation

#### 4.3.1. Histological Staining Procedures 

The animals were euthanized four weeks after the operation by intra-cardinal perfusion fixation with saline and 10% formaldehyde after deep anesthesia with Zoletil 50. The maxillary blocks were dissected and fixed in 10% buffered formalin for two weeks. After fixation, the samples were decalcified for four weeks in 0.1 M ethylene-diamine-tetraacetic acid, followed by washing, dehydration, embedding in paraffin, and serial sectioning at 4 μm in the sagittal plane from the mesiobuccal root using an automated rotary microtome (Leica RM2255, Leica Biosystems, Heidelberger, Germany). At each level, three sections were mounted, and the best section was selected for staining with hematoxylin and eosin (H&E).

Masson’s trichrome (MT) staining was performed to define bone and tooth resorption. This staining method typically produces red keratin and muscle fibers, blue or green collagen and bone, light red or pink cytoplasm, and dark brown to black cell nuclei. MT staining is often used in conjunction with H&E staining to provide a more comprehensive overview of the pulp and its cellular components, extracellular matrix, and adjacent dentine structure [33].

Enzyme histochemical tartrate-resistant acid phosphatase (TRAP) staining was utilized to detect osteoclast migration in a tooth with inflammatory root resorption, using a TRAP staining kit^®^ (Primary Cell Co. Ltd., Hokkaido, Japan). The staining method highlighted odontoclasts in bright pink color against the surrounding structures in pale purple and rose colors, clearly revealing the jagged dentine surface with clastic cells present in a resorption lacuna [33]. The slides were digitized using a digital slide scanner, Panoramic 250 Flash III (3DHistech, Ltd., Budapest, Hungary), and the final images were analyzed using CaseViewer software (version 2.0) (3DHistech, Ltd., Budapest, Hungary) to measure the areas of inflammatory and replacement resorption.

#### 4.3.2. Histological Analysis

Histological assessments of the periodontal healing of each tooth were evaluated in three categories by using H&E-stained specimens, and the descriptions of each category are as follows:

▪ Inflammatory root resorption: loss of the root surface with multinucleated inflammatory cell infiltration
▪ Replacement root resorption (ankylosis): loss of PDL cells with the direct fusion of bone tissue to cementum.
▪ Total root resorption: the sum of inflammatory and replacement root resorption considering that the two resorption patterns are completely different.

Inflammatory and replacement root resorptions were evaluated as a percentage of the total root length versus the length of the resorbed root on the basis of the mesiobuccal root treated with root canal treatment. The amount of each resorption was scaled from Grades 0 to 5 by modifying the method of a previous study [32]: Grade 0, not observed; Grade 1, less than 25% observed; Grade 2, less than 50% observed; Grade 3, less than 75% observed; Grade 4, more than 75% observed; and Grade 5, tooth exfoliation (Table 3). The average grades of the two examiners for each parameter were recorded. The analysis was performed by two experienced and trained examiners who were blinded to the group allocation. To assess inter-examiner consistency, intra-class coefficient values were calculated for the aforementioned parameters. The Cohen’s Kappa values indicated acceptable inter-examiner reliability (0.748–0.898).

### 4.4. Statistical Analysis

The histological results of each group were expressed as mean ± standard deviation, and data were analyzed using SPSS software (version 20.0) (SPSS Inc., Chicago, IL, USA). Comparisons were analyzed statistically using the Kruskal–Wallis test and Mann–Whitney test with post hoc analysis using Bonferroni correction. A *p*-value of 0.05 was considered statistically significant.

## Figures and Tables

**Table 1:** Histologically assessed mean values (±SD) of healing process according to groups.

	Group 1 (No Treatment)	Group 2 (NaF)	Group 3 (NO Gel)	Group 4 (DN Gel)
Inflammatory root resorption	2.50 ± 0.55 ^a^	1.67 ± 0.52 ^a^	2.00 ± 0.63 ^a^	1.50 ± 0.84 ^a^
Replacement root resorption	1.50 ± 1.05 ^a^	0.33 ± 0.52 ^a^	1.17 ± 0. 98 ^a^	0.67 ± 0.82 ^a^
Total root resorption	4.00 ± 0.63 ^a^	2.00 ± 0.63 ^b^	3.17 ± 0.75 ^ab^	2.17 ± 0.98 ^b^

Different case letters within the same rows indicate statistically significant differences, Kruskal–Wallis test and post hoc test with Mann–Whitney U-test corrected by Bonferroni correction.

**Table 2:** Experimental groups according to root conditioning agent.

	Root Conditioning Agent	Number
Group 1	No treatment	6
Group 2	2% NaF	6
Group 3	NO gel (10 mL)	6
Group 4	Doxycycline (1 mg) + NO gel (10 mL)	6
Total		24

**Table 3:** Histological parameters for periodontal healing events.

Grade	Inflammatory Root Resorption	Replacement Root Resorption	Total Root Resorption
0	Not observed		Sum of inflammatory and replacement root resorption
1	Less than 25% observed (<25%)		
2	Less than 50% observed (<50%)		
3	Less than 75% observed (<75%)		
4	More than 75% observed (≥75%)		
5	Tooth exfoliation		

## Data Availability

All data and materials are available on request from the corresponding author. The data are not publicly available due to ongoing researches using a part of the data.

## References

[1] Andreasen J.O., Andreasen F.M., Andersson L. (2013). Textbook and Color Atlas of Traumatic Injuries to the Teeth.

[2] McIntyre J.D., Lee J.Y., Trope M., Vann W.F. (2007). Management of avulsed permanent incisors: A comprehensive update. Pediatr. Dent..

[3] Fuss Z., Tsesis I., Lin S. (2003). Root resorption–diagnosis, classification and treatment choices based on stimulation factors. Dent. Traumatol..

[4] Andreasen J.O., Borum M.K., Jacobsen H.L., Andreasen F.M. (1995). Replantation of 400 avulsed permanent incisors. 4. Factors related to periodontal ligament healing. Endod. Dent. Traumatol..

[5] Choi S.C., Kwon Y.D., Kim K.C., Kim G.T. (2010). The effects of topical application of bisphosphonates on replanted rat molars. Dent. Traumatol..

[6] Selvig K.A., Bjorvatn K., Bogle G.C., Wikesjo U.M. (1992). Effect of stannous fluoride and tetracycline on periodontal repair after delayed tooth replantation in dogs. Scand. J. Dent. Res..

[7] Tsilingaridis G., Malmgren B., Skutberg C., Malmgren O. (2015). The effect of topical treatment with doxycycline compared to saline on 66 avulsed permanent teeth—A retrospective case-control study. Dent. Traumatol..

[8] Mori G.G., Nunes D.C., Castilho L.R., de Moraes I.G., Poi W.R. (2010). Propolis as storage media for avulsed teeth: Microscopic and morphometric analysis in rats. Dent. Traumatol..

[9] Mustafa M. (2017). Awareness about management of tooth avulsion among general dental practitioners: A questionnaire-based study. J. Orthod..

[10] Fouad A.F., Abbott P.V., Tsilingaridis G., Cohenca N., Lauridsen E., Bourguignon C., O’Connell A., Flores M.T., Day P.F., Hicks L. (2020). International Association of Dental Traumatology guidelines for the management of traumatic dental injuries: 2. Avulsion of permanent teeth. Dent. Traumatol..

[11] Yoo J.E., Kim M.S., Kwon Y.D., Kim E.C., Kim K.C., Choi S.C. (2015). Could zoledronic acid prevent root resorption in replanted rat molar?. Dent. Traumatol..

[12] Komatsu K., Shimada A., Shibata T., Shimoda S., Oida S., Kawasaki K., Nifuji A. (2008). Long-term effects of local pretreatment with alendronate on healing of replanted rat teeth. J. Periodontal Res..

[13] Alakangas A., Selander K., Mulari M., Halleen J., Lehenkari P., Mönkkönen J., Salo J., Väänänen K. (2002). Alendronate disturbs vesicular trafficking in osteoclasts. Calcif. Tissue Int..

[14] Aghaloo T.L., Hadaya D. (2017). Basic Principles of Bioengineering and Regeneration. Oral. Maxillofac. Surg. Clin. N. Am..

[15] Scheller E.L., Krebsbach P.H., Kohn D.H. (2009). Tissue engineering: State of the art in oral rehabilitation. J. Oral. Rehabil..

[16] Atala A. (2012). Regenerative medicine strategies. J. Pediatr. Surg..

[17] Granger D.N., Kubes P. (1996). Nitric oxide as antiinflammatory agent. Methods Enzym..

[18] Collin-Osdoby P., Rothe L., Bekker S., Anderson F., Osdoby P. (2000). Decreased nitric oxide levels stimulate osteoclastogenesis and bone resorption both in vitro and in vivo on the chick chorioallantoic membrane in association with neoangiogenesis. J. Bone Miner. Res. Off. J. Am. Soc. Bone Miner. Res..

[19] Sieber C.C., Sumanovski L.T., Stumm M., van der Kooij M., Battegay E. (2001). In vivo angiogenesis in normal and portal hypertensive rats: Role of basic fibroblast growth factor and nitric oxide. J. Hepatol..

[20] Kumar S., Singh R.K., Bhardwaj T.R. (2017). Therapeutic role of nitric oxide as emerging molecule. Biomed. Pharmacother..

[21] Backlund C.J., Sergesketter A.R., Offenbacher S., Schoenfisch M.H. (2014). Antibacterial efficacy of exogenous nitric oxide on periodontal pathogens. J. Dent. Res..

[22] Jun H.W., Paramonov S.E., Hartgerink J.D. (2006). Biomimetic self-assembled nanofibers. Soft Matter.

[23] Hubbell J.A. (2003). Materials as morphogenetic guides in tissue engineering. Curr. Opin. Biotechnol..

[24] Ban K., Park H.J., Kim S., Andukuri A., Cho K.W., Hwang J.W., Cha H.J., Kim S.Y., Kim W.-S., Jun H.-W. (2014). Cell therapy with embryonic stem cell-derived cardiomyocytes encapsulated in injectable nanomatrix gel enhances cell engraftment and promotes cardiac repair. ACS Nano.

[25] Lim D.J., Antipenko S.V., Anderson J.M., Jaimes K.F., Viera L., Stephen B.R., Bryant S.M., Yancey B.D., Hughes K.J., Cui W. (2011). Enhanced rat islet function and survival in vitro using a biomimetic self-assembled nanomatrix gel. Tissue Eng. Part A.

[26] Alexander G.C., Vines J.B., Hwang P., Kim T., Kim J.A., Brott B.C., Yoon Y.S., Jun H.W. (2016). Novel Multifunctional Nanomatrix Reduces Inflammation in Dynamic Conditions in Vitro and Dilates Arteries ex Vivo. ACS Appl. Mater. Interfaces.

[27] Yun K.H., Ko M.J., Chae Y.K., Lee K., Nam O.H., Lee H.S., Cheon K., Choi S.C. (2021). Doxycycline-Loaded Nitric Oxide-Releasing Nanomatrix Gel in Replanted Rat Molar on Pulp Regeneration. Appl. Sci..

[28] Krasner P., Rankow H.J. (1995). New philosophy for the treatment of avulsed teeth. Oral. Surg. Oral. Med. Oral. Pathol. Oral. Radiol. Endod..

[29] Pohl Y., Filippi A., Kirschner H. (2005). Results after replantation of avulsed permanent teeth. II. Periodontal healing and the role of physiologic storage and antiresorptive-regenerative therapy. Dent. Traumatol..

[30] Khademi A., Saei S., Mohajeri M., Mirkheshti N., Ghassami F., Torabi nia N., Alavi S.A. (2008). A new storage medium for an avulsed tooth. J. Contemp. Dent. Pract..

[31] Martins W.D., Westphalen V.P.D., Westphalen F.H. (2004). Tooth replantation after traumatic avulsion: A 27-year follow up. Dent. Traumatol..

[32] Gjertsen A.W., Stothz K.A., Neiva K.G., Pileggi R. (2011). Effect of propolis on proliferation and apoptosis of periodontal ligament fibroblasts. Oral. Surg. Oral. Med. Oral. Pathol. Oral. Radiol. Endod..

[33] Khinda V.I., Kaur G., Brar G.S., Kallar S., Khurana H. (2017). Clinical and Practical Implications of Storage Media used for Tooth Avulsion. Int. J. Clin. Pediatr. Dent..

[34] Poi W.R., Sonoda C.K., Martins C.M., Melo M.E., Pellizzer E.P., Mendonça M.R., Panzarini S.R. (2013). Storage media for avulsed teeth: A literature review. Braz. Dent. J..

[35] Andreasen J.O. (1981). Effect of extra-alveolar period and storage media upon periodontal and pulpal healing after replantation of mature permanent incisors in monkeys. Int. J. Oral Surg..

[36] Cvek M., Granath L.E., Hollender L. (1974). Treatment of non-vital permanent incisors with calcium hydroxide. 3. Variation of occurrence of ankylosis of reimplanted teeth with duration of extra-alveolar period and storage environment. Odontol. Rev..

[37] Shulman L.B., Kalis P., Goldhaber P. (1968). Fluoride inhibition of tooth-replant root resorption in Cebus monkeys. J. Oral. Ther. Pharmacol..

[38] Coccia C.T. (1980). A clinical investigation of root resorption rates in reimplanted young permanent incisors: A five-year study. J. Endod..

[39] Flores M., Andreasen J., Bakland L., Feiglin B., Gutmann J., Oikarinen K., Ford T.R., Sigurdsson A., Trope M., Vann W.F. (2001). Guidelines for the evaluation and management of traumatic dental injuries. Dent. Traumatol..

[40] Trope M. (2002). Clinical management of the avulsed tooth: Present strategies and future directions. Dent. Traumatol..

[41] Finucane D., Kinirons M.J. (2003). External inflammatory and replacement resorption of luxated, and avulsed replanted permanent incisors: A review and case presentation. Dent. Traumatol..

[42] Trope M., Moshonov J., Nissan R., Buxt P., Yesilsoy C. (1995). Short vs. long-term calcium hydroxide treatment of established inflammatory root resorption in replanted dog teeth. Dent. Traumatol..

[43] Consolaro A. (2002). Dental Resorptions in the Clinic Specialties.

[44] Lindskog S., Hammarström L. (1980). Evidence in favor of an anti-invasion factor in cementum or periodontal membrane of human teeth. Eur. J. Oral. Sci..

[45] Keum K.-Y., Kwon O.-T., Spängberg L.S., Kim C.-K., Kim J., Cho M.-I., Lee S.J. (2003). Effect of dexamethasone on root resorption after delayed replantation of rat tooth. J. Endod..

[46] Panzarini S.R., de Carvalho A.C.P., Poi W.R., Sonoda C.K. (2005). Use of vitamin C in delayed tooth replantation. Braz. Dent. J..

[47] Sorensen R.G., Polimeni G., Kinoshita A., Wozney J.M., Wikesjö U.M. (2004). Effect of recombinant human bone morphogenetic protein-12 (rhBMP-12) on regeneration of periodontal attachment following tooth replantation in dogs: A pilot study. J. Clin. Periodontol..

[48] Poi W.R., Carvalho R.M., Panzarini S.R., Sonoda C.K., Manfrin T.M., Rodrigues T.d.S. (2007). Influence of enamel matrix derivative (Emdogain^®^) and sodium fluoride on the healing process in delayed tooth replantation: Histologic and histometric analysis in rats. Dent. Traumatol..

[49] Dimmeler S., Zeiher A.M. (1999). Nitric oxide-an endothelial cell survival factor. Cell Death Differ..

[50] Hammarström L., Blomlöf L., Feiglin B., Andersson L., Lindskog S.J.D.T. (1986). Replantation of teeth and antibiotic treatment. Endod Dent Traumatol..

[51] Cvek M., Cleaton-Jones P., Austin J., Lownie J., Kling M., Fatti P. (1990). Effect of topical application of doxycycline on pulp revascularization and periodontal healing in reimplanted monkey incisors. Endod. Dent. Traumatol..

[52] Preshaw P.M., Hefti A.F., Jepsen S., Etienne D., Walker C., Bradshaw M.H. (2004). Subantimicrobial dose doxycycline as adjunctive treatment for periodontitis: A review. J. Clin. Periodontol..

[53] Ramamurthy N., Golub L. (1983). Diabetes increases collagenase activity in extracts of rat gingiva and skin. J. Periodontal Res..

[54] Ahn H.J., Nam O.H., Lee H.S., Kim E.C., Cohenca N., Choi S.C. (2016). Expression of inflammatory cytokines and MMPs on replanted teeth at different extra-alveolar time: An ex vivo and in vivo study. Int. J. Paediatr. Dent..

[55] Franco G.C., Kajiya M., Nakanishi T., Ohta K., Rosalen P.L., Groppo F.C., Ernst C.W., Boyesen J.L., Bartlett J.D., Stashenko P. (2011). Inhibition of matrix metalloproteinase-9 activity by doxycycline ameliorates RANK ligand-induced osteoclast differentiation in vitro and in vivo. Exp. Cell Res..

[56] Shulman L., Gedalia I., Feingold R. (1973). Fluoride concentration in root surfaces and alveolar bone of fluoride-immersed monkey incisors three weeks after replantation. J. Dent. Res..

[57] Kaushik S.N., Scoffield J., Andukuri A., Alexander G.C., Walker T., Kim S., Choi S.C., Brott B.C., Eleazer P.D., Lee J.-Y. (2015). Evaluation of ciprofloxacin and metronidazole encapsulated biomimetic nanomatrix gel on *Enterococcus faecalis and Treponema denticola*. Biomater. Res..

[58] Rambhia K.J., Ma P.X. (2015). Controlled drug release for tissue engineering. J. Control. Release.

